# The introduction of a paediatric nutrition support program led by a clinical dietitian at a low-resource hospital setting in Malawi

**DOI:** 10.1080/16549716.2019.1656452

**Published:** 2019-09-12

**Authors:** Allison I. Daniel, Humphrey Chatenga, Bernadette Chimera, Emmie Mbale, Mphatso Chisala, Eric Borgstein, Josephine Langton, Carmen Gonzalez, Robert H. J. Bandsma, Laura Vresk

**Affiliations:** a Centre for Global Child Health, Hospital for Sick Children, Toronto, Ontario, Canada; b Division of Gastroenterology, Hepatology and Nutrition, Hospital for Sick Children, Toronto, Ontario, Canada; c Department of Nutritional Sciences, Faculty of Medicine, University of Toronto, Toronto, Ontario, Canada; d Department of Paediatrics, Queen Elizabeth Central Hospital, Blantyre, Malawi; e School of Public Health and Family Medicine, College of Medicine, University of Malawi, Blantyre, Malawi; f Department of Surgery, College of Medicine, Blantyre, Malawi; g Malawi-Liverpool Wellcome Trust Clinical Research Programme, Blantyre, Malawi; h Department of Biomedical Sciences, College of Medicine, University of Malawi, Blantyre, Malawi; i Department of Clinical Dietetics, Hospital for Sick Children, Toronto, Ontario, Canada

**Keywords:** Nutritional status, nutritional support, dietetics, quality of care, qualitative research

## Abstract

In low- and middle-income countries, nutrition support strategies are often suboptimal or non-existent in hospital settings. This is further compounded by high rates of malnutrition in these countries. The first four dietitians graduated in Malawi in 2017 providing a new opportunity to build capacity to introduce nutrition support in an acute care setting. A paediatric nutrition support program was implemented at Queen Elizabeth Central Hospital (QECH) in Blantyre, Malawi including the hiring of a local dietitian. This capacity building paper explains the development and introduction of the nutrition support program including a description of perceptions of health professionals at QECH working alongside the dietitian. In the first four months of the program at QECH, the dietitian provided nutrition support to 183 different patients across paediatric wards. Nutritional interventions predominantly included infant formula and breastmilk fortification, provision of therapeutic feeds orally or via nasogastric tubes, increased dietary protein intake for children identified to be at high risk, and nutritional counselling to caregivers. More complex nutritional interventions were also given such as the insertion of gastrostomy tubes to deliver nutrition directly to the stomach. Following the introduction of the program, qualitative interviews were done with health professionals at QECH including nurses (n = 5) and physicians (n = 11). All participants emphasized the importance and impact of the nutrition support program in enhancing the care of hospitalized children, therefore improving outcomes such as tolerability of clinical interventions, decreased duration of stay, and reduced risk of hospital readmission. In conclusion, there is a need for nutrition support provided by a dietitian for different paediatric patients which was corroborated by positive feedback from health professionals at QECH. Integration of dietitians into the healthcare system by respective Ministries of Health will require advocacy around the potential for nutrition support to strengthen the quality of care of vulnerable children.

A Chichewa abstract for this paper is available in a supplementary file.

## Background

Achieving universal health coverage is a major objective of the World Health Organization (WHO) [1]. Malawi is a low-income country that is aiming to achieve universal health coverage by 2030 [2,3]. One of the mandates of universal health coverage is that health services should be of high enough quality to improve health outcomes, with the most vulnerable people being prioritized [1]. Recent evidence has shown that poor quality care is a greater contributor to mortality than insufficient access to care [4]. A key component of quality of care is the health workforce; Sustainable Development Goals Target 3.c. focuses on increasing ‘recruitment, development, training and retention of the health workforce in developing countries, especially in least developed countries’ [5].

The 2015–16 Malawi Demographic and Health Survey showed that 37% of children are stunted, 3% are wasted, and 12% are underweight, while 63% of children are anaemic [6]. In hospitalized children, poor nutritional status is associated with inpatient and post-discharge morbidity and mortality, yet nutrition support for these children is extremely limited with few qualified individuals to provide nutritional interventions in low-resource settings like Malawi [7–11]. It is therefore evident that there is a need for clinical nutrition support in low-resource hospital settings to improve the quality of care of vulnerable children. In high-income settings, nutrition support is an integral part of care for treating a wide range of paediatric patients [12–18]. Nutrition support teams include dietitians, clinicians, and other health professionals involved in clinical care of patients. Importantly, dietitians’ roles typically include nutritional assessment, estimation of nutritional requirements, and provision of nutritional interventions with close monitoring of patients. This generally is followed by nutritional counselling at the time of discharge from hospital.

The first four locally trained dietitians in Malawi graduated from the accredited post-graduate clinical dietetics program at the Lilongwe University of Agriculture and Natural Resources (LUANAR) in Lilongwe, Malawi in 2017 [19]. One of the dietitians was recruited to work in the Department of Paediatrics at Queen Elizabeth Central Hospital (QECH) in Blantyre, Malawi. In this capacity building paper, we describe the introduction of a clinical nutrition support program at QECH, led by a newly-trained local dietitian entering the health workforce. We aimed to further understand the phases before the implementation of nutrition support, during the introduction of the program, and the future of the program by undertaking qualitative interviews with health professionals at QECH who worked alongside the dietitian.

## Context of the nutrition support program

QECH is a tertiary care center that acts as the main teaching hospital for the University of Malawi College of Medicine. Within the Department of Paediatrics at QECH, approximately 26000 children are treated as inpatients each year with a mortality rate of around 5% [20,21]. Paediatric patients are triaged at the paediatric emergency department and can then be admitted to the paediatric intensive care unit (PICU), surgical ward, special care ward, nutritional rehabilitation unit (NRU), medical bay, oncology ward, or nursery. Patients with burns are directly admitted into the burns unit, which treats both adults and paediatric patients.

Upon presentation to the paediatric emergency department of QECH, children may not be routinely screened for malnutrition and anthropometry is not typically recorded on the admission sheets or inpatient files with the exception of weight [20,22]. Furthermore, one previous study of 53 nurses at QECH showed that 71% of clinical charts of patients who were fed through nasogastric tubes (NGTs) had no documentation of nutritional assessment, and it was self-reported by 43% of nurses that they felt they were not competent in nutritional assessment [20,22]. Nurses also stated that communication about nutritional care with clinicians is commonly done by only half of the nurses [22]. However, just 3% of clinical charts had documentation of written communication about nutritional care with clinicians [22]. Additional qualitative interviews with health professionals involved in the care of critically ill patient at QECH and another tertiary hospital in Lilongwe, Malawi have indicated that training and protocols around nutrition support, apart from protocols on severe acute malnutrition (SAM), are lacking and there are few qualified individuals to lead nutrition support including clinical nutritionists and dietitians [9]. Collectively, these findings suggest that there is a gap in providing nutrition support for paediatric patients within this low-resource hospital setting.

## Building local capacity for nutrition support

The induction of the LUANAR clinical dietetics program was the first step in the integration of dietitians into the health workforce in Malawi. The following sections of this manuscript focus on experiences with one Malawian dietitian who graduated from this program. A one-year contract for the dietitian was supported by the Catalyst Grant from the Centre for Global Child Health at the Hospital for Sick Children (SickKids) in Toronto, Canada.

An experienced SickKids dietitian conducted a two-week site visit in Malawi prior to the introduction of the nutrition support program. A needs assessment was done in conjunction with local physicians, including the head of the Department of Paediatrics, to plan the implementation of the nutrition support program at QECH. The SickKids dietitian also attended daily clinical handover and ward rounds to gain a stronger understanding of the context. Based on this assessment, all aforementioned wards with paediatric inpatients at QECH were selected to be part of the program. The newborn intensive care unit is an additional paediatric ward at QECH which was considered too large to be managed by one dietitian in addition to the other wards, and therefore was omitted from the program.

Experts from SickKids, including dietitians, nutritionists, and clinicians were available to provide mentorship to the local dietitian in Malawi upon the start of the nutrition support program. This included on-the-ground training with the SickKids dietitian for the first 3 weeks of work at QECH beginning in August 2018. The SickKids dietitian accompanied the local dietitian for ward rounds during these 3 weeks and identified various patients to whom nutrition support could be provided. This included a detailed nutrition assessment and plans for each individual patient. During this orientation, the dietitians met with health professionals at each of the respective wards and involved them in care of these patients to familiarize them with nutrition assessment and interventions. In addition to providing care to individual patients, basic nutrition protocols were developed for the different wards at the hospital.

After these initial 3 weeks, the local dietitian began ward rounds and patient consults as the sole dietitian at QECH. The local dietitian worked with health professionals across paediatric wards including nurses, physicians, and clinical officers. As the lead of the nutrition support team, the dietitian interacted with these personnel to emphasize the importance of nutrition support, to identify high-risk patients who could benefit from specific interventions, and to involve all health professionals in the delivery of interventions and monitoring of children receiving nutritional support. Bi-weekly nutrition rounds via Skype with the SickKids dietitian were held for case presentations in which the local dietitian identified two complex cases, discussed nutrition concepts and considerations relevant to these cases, and created plans for nutrition support for these patients. Examples of cases were: an adolescent with pathological fractures requiring feeding through a NGT in addition to vitamin D and calcium supplements; an infant undergoing a colostomy needing breastmilk fortification; and a child with an acquired trachea-oesophageal fistula necessitating a gastrostomy tube. In addition, the SickKids dietitian was consulted by the Malawian dietitian to discuss challenging cases outside of these planned rounds via phone applications. This approach was to allow the dietitian to provide nutrition support that was feasible within this context.

## Implementation of nutrition support

The main elements of the nutrition support program at QECH included provision of enteral feeds, fortification of expressed breastmilk, and other nutritional interventions such as distribution of therapeutic feeds, as well as counselling of caregivers upon discharge from hospital to promote nutritional recovery following inpatient treatment. The local dietitian attended the daily clinical handover for the entire paediatric service prior to doing rounds across each of the eight wards throughout the week. Following ward rounds, the dietitian completed consults across wards according to specific patient needs and referrals. A record of consults was kept by the dietitian as part of routine care to document nutrition assessment and classification of malnutrition, clinical and nutritional reasons for requiring nutrition support, and types of nutritional interventions provided.

In 4 months, from August to December 2018, the dietitian provided nutrition support to 183 different paediatric patients admitted to QECH for treatment of chronic conditions, trauma including burns, and acute illnesses and infections. Of these, 111 children who were not already admitted to the nutritional rehabilitation unit were identified to have SAM and 16 children were identified to have moderate acute malnutrition (MAM) according to the WHO Child Growth Standards [23]. This is a high number of malnourished children who were not identified upon admission to hospital.

One intervention that was provided frequently as part of the nutrition support program was the fortification of infant formula or expressed breastmilk, which was predominantly done by the dietitian. In preparation for cases where the dietitian was unavailable, recipes and instructions for fortification were placed in all different wards and education was provided to the head nurses of each of the wards. Therapeutic feeds, including F-75, F-100, and Ready-to-Use Therapeutic Foods, were given to acutely malnourished children either orally or via NGT [23,24]. Children eligible for admission for inpatient treatment of SAM were transferred to the NRU unless they required specific care in certain wards such as the burns unit [23]. Upon discharge from hospital, any children that had acute malnutrition were referred to outpatient feeding programs as per the WHO guidelines for Community-based Management of Acute Malnutrition [25]. The dietitian also gave nutrition counselling to caregivers at the time of discharge from the hospital. Counselling included tailored messages such as how to control carbohydrate intake for diabetic patients who were admitted with uncontrolled blood sugar levels or sources of dietary protein for children with severe burns with increased nutritional requirements.

There were several challenges encountered by the dietitian during the implementation of this program mainly due to the limited resources available for nutrition support (Table 1). However, even with restricted feeds, equipment, and personnel, nutrition support could be provided in this low-resource setting by applying simple solutions that were developed by the dietitian at QECH based on what was generally most effective for common situations in this context.

**Table 1.** Challenges encountered and solutions offered by the dietitian during the first 4 months of the nutrition support program at Queen Elizabeth Central Hospital.

Challenges	Solutions
High number of patients requiring nutrition support	Engage other health professionals who can conduct nutrition assessment and provide interventions such as expressed breastmilk and formula fortification
Inadequate supply of nutrition products	Use existing therapeutic feeds and fortify expressed breastmilk and formula that is available
Difficulty in monitoring exact intake and losses	Subjectively rely on guardian recall upon explaining to guardians the importance of this and how to assess intake and losses
Poor communication with dietitian when discharging patients	Add label in patient files to call dietitian at the time of discharge for counselling
Inadequate time to follow up children receiving outpatient treatment after discharge	Review specific outpatients such as those with feeding difficulties upon referral

## Perceptions of health professionals

Qualitative interviews were undertaken to describe the phases before the implementation of the nutrition support program, during the introduction of the program, and the future of the program from the perspective of health professionals. Participants were selected by a clinician within the Department of Paediatrics at QECH (CG) through purposive sampling with a goal of recruiting two health professionals for each of the eight wards, as it is common that there is one head nurse and one physician per ward at minimum. Interviews were held with nurses (n = 5) and physicians (n = 11) at QECH in November and December 2018. None of the eligible participants declined to participate in interviews.

Consent procedures and interviews were completed in English, which all participants are fluent in, in private settings at QECH by a researcher with experience in qualitative methods (AID). This researcher is not involved in patient care at QECH. A semi-structured interview guide was used and included questions pertaining to previous nutrition support or interventions provided before the implementation of the program, perceived importance of nutrition support for paediatric patients, if and how working with a dietitian has changed care of these patients, barriers to providing nutrition support in this setting, and how to sustain this type of program in the future. Interviews, which lasted approximately 15 min on average, were audio-recorded. The researcher also documented ideas about potential themes that arose during the interviews [26].

Data were de-identified during the transcription process and were presented without information of participants, including the type of health professionals, to avoid revealing their identities. Thematic analysis was undertaken to analyze the qualitative data [26,27]. An initial coding framework from all interviews was developed inductively by one researcher (AID) using NVivo 11 software and was discussed further with a second investigator (LV) to then come to a consensus on the categorization, refinement, and description of themes supported by specific participant quotations [26–28]. The Standards for Reporting Qualitative Research were used in the description of this qualitative study [29]. Ethics approval was obtained from the College of Medicine Research and Ethics Committee and the SickKids Research Ethics Board.

There were five themes that emerged from the qualitative interviews which were categorized by phases around the introduction of the nutrition support program (Table 2).

**Table 2.** Themes arising from qualitative interviews of health professionals at Queen Elizabeth Central Hospital.

Themes	Phases
Lack of nutrition support prior to the introduction of the nutrition support program	Before the implementation of the nutrition support program
Overall significance of introducing the nutrition support program	During the introduction of the nutrition support program
Receptiveness of guardians to the nutrition support program	During the introduction of the nutrition support program
Involvement of health professionals in the nutrition support program	During the introduction of the nutrition support program
Sustainability of nutrition support in low-resource hospital settings in Malawi	Future of the nutrition support program

The first theme identified from the interviews described the lack of nutrition support across wards at QECH prior to the introduction of the nutrition support program. A majority of participants stated that this meant relying on the NRU whenever possible.

The only nutritional support was the [nutritional rehabilitation unit] for malnourished children. But for the patients in the other wards, without diagnosis of nutrition issues, let’s say acute malnutrition in surgical cases, nutrition support was non-existent. But there are some we really need to optimise nutritionally before we operate.

The following three themes related to the introduction of the nutrition support program. Specifically, the second theme was about the overall significance of providing nutrition support and having a dietitian in this low-resource setting, which all participants stated enhances the care that they can provide. Participants highlighted this with specific examples of positive outcomes in patients receiving nutrition support since the introduction of the program. According to participants, patients who have benefitted from nutrition support are children with burns, typhoid perforations, hydrocephalus, pneumonia, acute malnutrition, cerebral palsy (CP), and cancer.Outcomes that participants subjectively stated that they observed were weight gain, improved wound healing, shorter duration of hospital stay, and reduced risk of readmission or death.

So far, we have seen that patients that are getting nutritional support have better outcomes in terms of recovery from cancer and even tolerability; they can tolerate chemotherapy better than malnourished ones. It changes how long they stay in the hospital. A patient that is malnourished will delay to complete treatment, but a patient who has good nutrition can go through chemo quickly compared to malnourished ones.

There was one participant who stated that though nutrition support improved outcomes in most patients, in a few cases in which nutrition support was not successful it gave clinicians another perspective.

For the babies who we have not been successful with, it has prompted us to look for other things. There were some babies that came in and we thought that it was purely feeding issues. We found out that they were receiving maximum feeding support and they were still not improving. So, it has helped us widen our differentials, while in the past we assumed that mothers weren’t doing something right.

Thirdly, some participants discussed the perspectives of guardians of children who received nutrition support and specifically their receptiveness to it.

Dietetics sometimes incorporates the guardians into the care. That has got a lot of benefits. That guardian is likely to carry on doing what she learned in the hospital at home. It’s not increasing burden on the nurses, but it only harnesses the care that the guardians provide to the kids.

However, one participant explained that there could be resistance to certain nutritional interventions such as the insertion of NGTs which could be perceived by guardians to be associated with negative outcomes like mortality.

Critical patients who get interventions such as nasogastric tube feeds, oxygen therapy, there’s a perception that those interventions lead to death but it’s because they are so critically ill that they get the interventions. So, guardians and patients sometimes refuse interventions because of that association. We would like to educate the patients and the guardians about these interventions and to show them what difference they can make.

Another theme that arose was around involvement of health professionals aside from the dietitian in nutrition support. One message discussed by most participants was about how to increase health professionals’ awareness and knowledge of nutrition.

We have always wanted a dietitian so when he came it was a good thing and we asked that he also teach the staff in the burns unit, so the staff have been educated. We did some tutorials previously, but it needs someone to actually spearhead the initiatives like this. Otherwise, people have the knowledge without having the materials or without someone having a keen interest. We just do the routine and we don’t really give the nutritional support the patients need. I think he gave one or two talks, like continuous professional development for all the staff in the unit.

Some participants articulated that although involvement of other health professionals in nutrition support is necessary, having a dietitian can reduce workload and allow for them to focus on their main clinical roles.

Once I see that a patient needs nutrition support, I don’t even have to think about it to say I should calculate, I should do the basic calculations that I used to do which aren’t the right ones. Now all I do is I just say can you please tell the dietitian to come see this child. It has reduced my workload and I am able to concentrate more on my proper surgical part.

One participant also stated that the dietitian has connected health professionals from across wards at QECH as he is one of the few people treating patients across different wards at the hospital.

I think that of course the dietitian has specific knowledge that is very useful, but I think that the most important part is that he has united all of us. Previously there was no communication at all and I could go to someone in a different ward and ask for something and it was always a no. Through him, if I would go and say consider a gastrostomy tube, for example, then they consider it. It’s linking everything together.

The last theme that developed from the interviews was concerning the sustainability of nutrition support in the future. Several participants described the challenge of ensuring a consistent supply of feeds for nutrition support. Many participants also emphasized the importance of support from the Ministry of Health to retain dietitians at hospitals in Malawi.

Any sane Ministry [of Health] would recognise that this is part of care and therefore the initiative must be supported. When we are treating patients, you don’t only look at the aspect of drugs. You must look at the whole patient, nutrition inclusive.

## Future directions

This capacity building project focuses on nutrition support provided by one dietitian at QECH who was supported by experts at SickKids. However, as described previously, nutrition support teams in high-income settings typically include health professionals, aside from dietitians, such as feeding therapists. The qualitative interviews undertaken highlighted a theme about the involvement of other health professionals in nutrition support, including the importance of understanding and having the capacity to provide nutrition interventions. Participants in the qualitative study also discussed that the provision of some nutrition interventions requires collaboration with different health professionals across wards. Evidently, further steps must be taken to increase the capacity of all health professionals to provide nutrition support at QECH.

Moving forward with the nutrition support program at QECH, there is also potential for more complex nutritional interventions as a new PICU and surgical unit opened in 2017. For example, the surgical insertion of gastrostomy tubes through the abdominal wall allows for direct delivery of food into the stomach. This type of intervention can also benefit specific patient populations such as children with SAM and CP. Six per cent of malnourished children admitted to the NRU at QECH have CP or other disabilities associated with feeding intolerance and swallowing impairment [30]. A next step at the PICU and surgical unit at QECH could also be to initiate total parenteral nutrition (TPN), a feeding method that involves the insertion of central venous catheters (CVLs) to circumvent the gastrointestinal tract. TPN is commonly provided as standard treatment for children in PICUs in high-income countries, though it is not typically applied in low-resource settings. TPN requires maintenance of CVLs to minimize infection risk, which is particularly important in this setting, in addition to close monitoring of feeding and nutritional status of the patients.

At a national level, integrating more dietitians into the healthcare system in Malawi is an overarching priority which was also highlighted in the qualitative interviews. A call for dietitians to begin work at central hospitals in Malawi was announced in December 2018. Only two low-income Sub-Saharan African countries, Ghana and Nigeria, have published literature about the practice of dietitians, both of which have limited training programs for dietitians and insufficient continued support for dietitians entering the health workforce [31,32]. Malawi could therefore act as a leader in enhancing the quality of care with nutrition support in the context of universal health coverage in low- and middle-income countries. Successful implementation will also require resource allocation for feeds and other supplies for nutrition support, anthropometry equipment, and training of other health professionals as part of nutrition support teams.

## Conclusions

During the introduction of a nutrition support program at this low-resource hospital setting, one dietitian provided nutritional interventions to 183 patients; this would total around 550 children annually. Qualitative interviews with health professionals working alongside the dietitian also emphasized the potential for nutrition support to improve overall quality care of vulnerable children in this context. Ultimately, there is an urgent need for Ministries of Health to support dietitians entering the health workforce in low- and middle-income countries globally.

## Paper context

A paediatric nutrition support program led by a locally trained dietitian was introduced at Queen Elizabeth Central Hospital in Blantyre, Malawi. The dietitian provided nutrition support to 183 children across eight wards in the first 4 months of the program. Qualitative interviews with nurses and physicians highlighted the value of nutrition support to improve quality of care in low-resource hospital settings.
