# Supplementary material for: The introduction of a paediatric nutrition support program led by a clinical dietitian at a low-resource hospital setting in Malawi
Source: Glob Health Action. 2019 Sep 12;12(1):1656452. doi: 10.1080/16549716.2019.1656452 (PMC6746265; doi:10.1080/16549716.2019.1656452)
Supplement: Supplemental Material [file ZGHA_A_1656452_SM8326.pdf]

Chichewa abstract for “The introduction of a paediatric nutrition support program led by a clinical dietitian at a low-resource hospital setting in Malawi”, Global Health Action 2019; 12:1656452, as supplied by the authors.

CHICHEWA ABSTRACT

Muzipatala za m'maiko osaukisitsa ndi m’maiko osaukilapo, chithandizo chokhudza madyedwe chimakhala chopelewera kapena sichipezeka nkomwe. M'maiko ngati amenewa, vutoli limaonjezereka ndi kuchuluka kwa vuto la kusowa kwa zakudya m’thupi. Madotolo a zamadyedwe anayi omwe anali oyambirira kuphunzitsidwa mu Malawi anamaliza maphunziro awo mu 2017. Ndondomeko ya kadyedwe ka ana inakhazikitsidwa pa chipatala cha Queen Elizabeth Central Hospital (QECH) mu mzinda wa Blantyre ku Malawi. Dokotala wa zakadyedwe analembedwa ntchito pa chipatalapo. Pepala ili likufotokoza za momwe ndondomeko ya kadyedweyi inayambira kuphatikizapo maganizo a anthu ogwira nchito ya zaumoyo pa chipatala cha QECH amene akugwira ntchito limodzi ndi dokotala wa kadyedweyu. Kuonjezera apo tinalembanso dotolo wa zakudya m’modzi. Mu miyezi inayi yoyamba chikhazikitsireni ndondomeko imeneyi pa QECH, dotolo wa za thanzi anapereka thandizo la madyedwe oyenera kwa odwala okwanira 183 ndi kwa ana ogonekedwa m'chipatalamo. Thandizo la madyedwe lomwe linaperekedwa linali monga, mkaka ogula wa ana, kuonjezera michere mu mkaka wa m’mawere, kuonjezera zakudya za mchiliso kudzera pakamwa kapena chubu champhuno, kuonjezera zakudya zolimbitsa thupi kwa ana ofunikira kutero, kupereka uphungu wa madyedwe kwa osamalira ana. Thandizo lapatsogolo lomwe limaperekedwa linali monga, kupereka zakudya kudzera muchubu cha m’mimba. Tinapanganso kafukufuku kuti timve maganizo kuchokera kwa anthu ogwira ntchito za umoyo pokhudza ndondomekoyi. Kafukufukuyu tinapanga ndi anamwino 5 ndi madotolo 11. Muzotsatira, a zaumoyo onse anatsindika za kufunika kwa ndondomeko yothandiza madyedwe kwa ana ogonekedwa mu chipatala kuti zinathandiza kupititsa patsogolo zotsatira za thandizo lomwe limaperekedwa kwa ana a pa chipatalapo. Pofotokoza, a zaumoyowa anaona kut matupi a anawa anali omasuka kulandira chithandizo, kuchepetsa nthawi yogenekedwa m'chipatala komanso kuchepetsa kugonekedwa m'chipatala mobwerezabwereza. Poomba nkota, ndondomeko ya kadyedwe ka ana, motsogozedwa ndi katswiri wa kadyedwe, ndiyofunikira kwambiri kwa ana osiyanasiyana omwe agonekedwa pa chipatalachi. Izi zili molingana ndi maganizo a akadaulo a za umoyo pa chipatala cha QECH. Kuti nthambi zosiyansiyana mu unduna wa zaumoyo zionjezere madotolo a zamadyedwe mu gulu la a ntchito a zaumoyo ogwira nchito pa chipatala pafunika kudziwitsa ndi kulimbikitsa anthu pa ubwino oonjezera ndondomeko yothandizira madyedwe kwa ana oti ali pachiopsezo.
